# Supplementary figures and images for: MAGIC: A tool for predicting transcription factors and cofactors driving gene sets using ENCODE data
Source: PLoS Comput Biol. 2020 Apr 6;16(4):e1007800. doi: 10.1371/journal.pcbi.1007800 (PMC7162552; doi:10.1371/journal.pcbi.1007800)

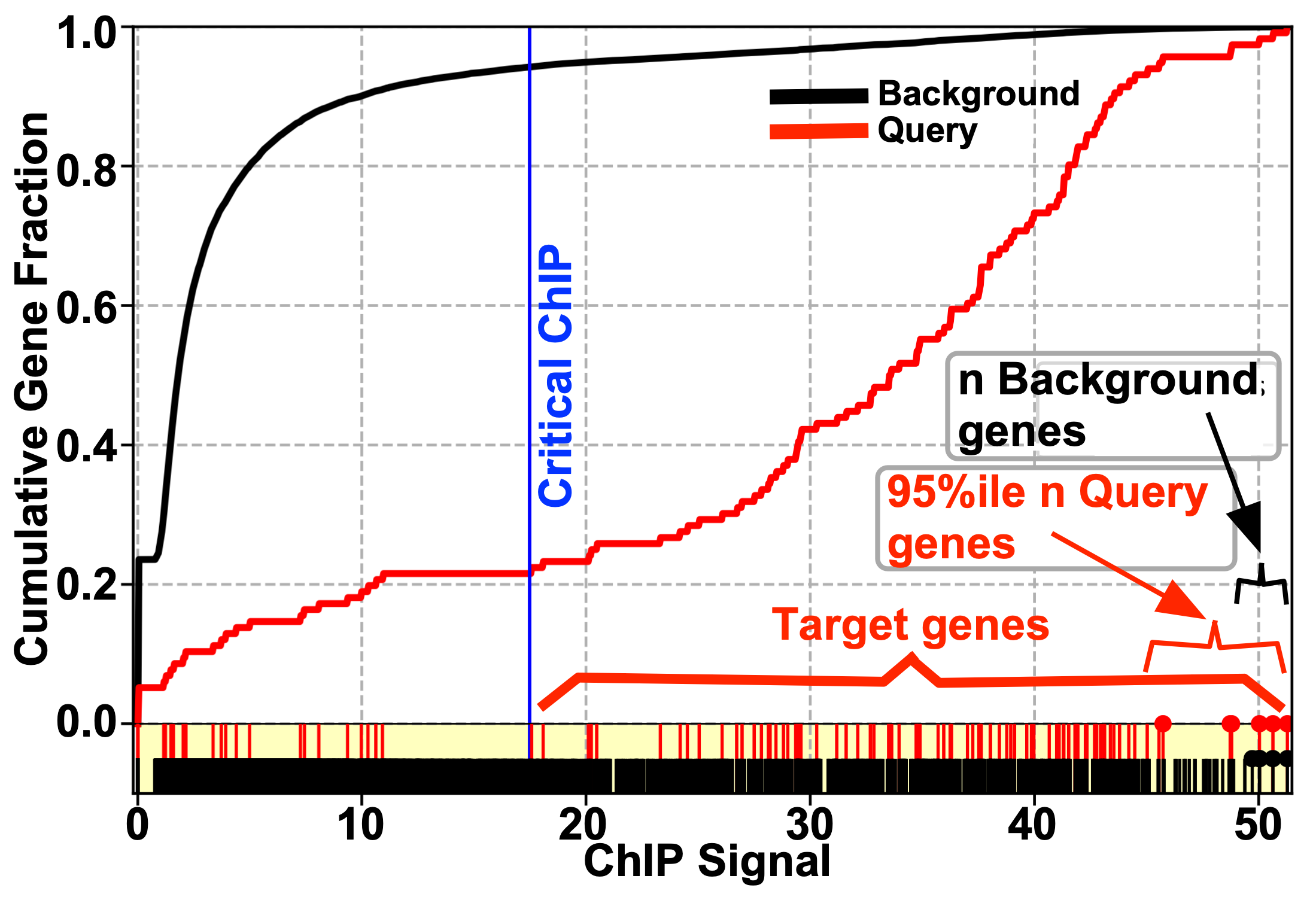

Supplement: S1 Fig — Two cumulative functions are displayed: the black curve is the fractional cumulative of all genes in the background list against ChIP values, red is the same for query genes. A blue vertical line denotes the ChIP value at Dsup i.e. argDsup. Red ticks along the x-axis represent each gene in the query list and black ticks are all genes in the background. Red ticks with circles (‘lollipops’) are the n (n = 0.05X) best chiped genes. Black lollipops are genes in the background list with the n highest ChIP values. (TIFF) [file pcbi.1007800.s001.tiff]
