# Supplementary material for: MAGIC: A tool for predicting transcription factors and cofactors driving gene sets using ENCODE data
Source: PLoS Comput Biol. 2020 Apr 6;16(4):e1007800. doi: 10.1371/journal.pcbi.1007800 (PMC7162552; doi:10.1371/journal.pcbi.1007800)
Supplement: Attachment — Submitted filename: Response_to_Reviewers.docx [file pcbi.1007800.s014.docx]

Reviewer #1:

Major (for me I think it is a Major revision but I will register this review only as minor revision because the validation of your algorithm can be done independently and later by other researchers)
1) Need validation of your algorithm: To be sure that your algorithm is interesting for life science research the validation of the prediction of the algorithm need to be done using 'wet lab' experiments. 'Cherry picking' online references is okay for 'discussion' but it not a validation of your algorithm.

I have added an extra dataset in addition to the three in the previous submission. Also, I cite a manuscript from my lab that utilized the prototype version of this algorithm and validated it's predictions. This is now discussed in the current resubmission.

Introduction

"We posit that in many cases, the majority of those changes are driven by alterations in the function of a few Factors that coordinate programmatic gene changes on a genome wide scale."
-> By opposition to clonal cell line, cancerous tissue is a mix of hundred different cell types. I think the observed change in gene transcript quantity is principally related to the change in cell quantity inside the sample. Please discuss this aspect of cell diversity in tissues analyzed and how this affects the observed changes in gene level quantity. e.g. : A disease tissues should have a drastic increase in the quantity of immune cells, Cancer tissues can show a significant change in the quantity of blood vessels...

I thank the reviewer for bringing this up as it was an oversite to not mention tissue heterogeneity in the original draft. The paragraph has been modified to state “*The changes reflect both alterations in tissue heterogeneity and alterations in transcriptional regulator function. We posit that in many cases, the majority of cellular transcriptome changes are driven by alterations in the function of a few Factors that coordinate gene programs. Identifying those driving Factors is a fundamental problem and therapeutic opportunity.”*

Methods
-> Explain RNA extraction protocol. Trizol is an old technique, why not using Qiagen RNeasy columns?

This is no longer relevant in the current submission as all datasets have been published and extraction protocols are described in cited publications.

"Probes were then collapsed to a single gene value ..."
-> 1 Gene with 1 value assigned to it is not what happen in reality. You need to talk about the problem of alternate splicing and multiple transcripts expressed per gene. Can the Transcription Factors have an influence on the complexity of RNA expression inside a single gene? Does the probes have been designed to target multiple transcripts per gene?

I agree that the question of assigning probes to genes and incorporating splice information is a very interesting and important topic. However, this whole section no longer exists in the new extensively revised version of the manuscript and a discussion of how TFs may contribute to the relative contribution of each splice form to the population of transcripts from a gene is beyond the scope of this article. I do however clarify how a gene is considered for analysis re: background lists for analysis. That is covered both in the Methods, Results and Discussion. I very much look forward to discussing details of TFs and splicing with you one day though!

"any genes changed more than 3 Standard Deviations from the mean fold change..."
-> please explain more your methods to compute fold change of each gene. Which standard deviation are you using, is it the Standard Deviation between Probes from the same gene or between factors?

I apologize for not being clearer about this earlier and have clarified the text. Thus: “*Genes up-regulated more than 3 Standard Deviations from the mean fold change (average shREST value/ average shCon value) and having a Benjamini Hochberg corrected p value<0.05 were used for Factor analysis.”*

"Sams et al profiling..."
-> What is the meaning of ‘Sams et al profiling’ ?.
-> Explain RNAseq protocol

Sams et al profiling refers to the RNAseq profiling described in the Sams et al publication (reference 17 in the manuscript). There is no longer an RNAseq protocol here as it is described in Sams et al.

"were assigned a gene domain that was defined as 5Kb either side of the gene body.."
-> Explain why you choose 5Kb, why not 1KB? reference needed. Else can you add an option to your algorithm to scan for gene domain with a shorter or larger window and then report the most significant with some sort of ‘weight’ based on distance? (e.g.: More it is close of the RNA start and more it is significant)

Thank you for raising this point. It was one of the key components of your review that led to a major change in the MAGIC algorithm. The user now has a choice of matrices to use for analysis. The user can use matrices containing binding data of Factors within a 5Kb window of the gene body, 1Kb window of the gene body, 1Kb of the promoter, with or without recombinant Factor data. Future versions of MAGIC will incorporate a dynamic domain window that will self-optimize to find the most impactful size for each Factor. Again, thanks for raising this as your question had a major impact on the redesign of the algorithm.


" = -log (corr) × "
-> Can you please explain the scientific validity of this type of scoring (multiplication of transformed significance by ratio)? Do you assume that the log of significance (corrected pvalue) and the ratio of means will follow a similar distribution?, if not I think this scoring can induce a bias in the detection of more significance (lowest pValue) or to the opposite to the detection of bigger ratio, all this will dependent of the shape of the corresponding distribution of ‘Pcorr’ and ‘r’. Will it be possible to adapt your algorithm and add an option to rank the genes by ‘r’ using a fixed corr (e.g.: p< 0.01? or even p< 0.1?)

Some Factors are associated with a low p or Pcorr but small effect size. Others have a large effect size (measured by either the Kolmogorov D statistic or tail ratio (Uq/Ub)) and higher p value. Multiplying the confidence (-log(p)) by a measure of effect size aims to allow prioritizing based on p value or effect size. The user is able to choose to prioritize by just p value by opening the summary.xls file and ordering results by p or FDR or Score.


Results
-> Please define "FDR", is it the False Detecting Rate? how do you compute it? is it the same as corr? If it is False Detecting Rate can you use ‘%’, (e.g. FDR 5% instead of FDR 0.05)

FDR is the False Discover Rate and is computed using the Benjamini-Hochberg correction. This is now explicitly stated in the manuscript. Thanks for suggesting this clarification.

-> ‘biologically meaningful’ can not be done by ‘cherry-picking’ online references or pathways because you will report only the reference that are validating your results and not the ones that go against. Your predicted results need to be validated by ‘wet lab’ experiments, what type of ‘wet lab’ experiments did you do to validate your informatics results?

The prototype that gave rise to the current algorithm was validated by wet lab analysis and the work is published in Khan et al, 2019 and is now discussed in the manuscript.


Discussion
-> How does your algorithm react when more than 1 gene share the 5Kb window? Do you have some way to reduce the significance in function of the number of genes that share this TF (e.g.: if using a 5Kb windows you found 4 genes that share the same TF, only 1 gene is of interest I think the significance of this TF should be reduced because you don’t know which gene this TF is affecting)

I applaud the reviewer’s thoughtful insight into these deep questions. The current version of the manuscript does not scale the ChIP value of a Factor if it lays between 2 close genes. I do not believe the field is yet in a position to model how such scaling will work. Thus, currently, if a Factor lays between 2 proximal genes, it will be assigned to both with equal weight. I assure the reviewer that we are working to develop a universal model to allow partitioning of Factor function amongst gene clusters and once we have such a model in hand, it will be incorporated into future MAGIC versions.


Reviewer #2: The manuscript by Avtar Roopra presents a useful tool MAGICTRICKS which identifies enriched transcription factors (TFs) whose signals are enriched in genes (5'UTR-5kb to 3'UTR+5kb) in a query gene list (e.g. differentially expressed genes) compared to a master gene list (e.g., expressed genes). The tool identifies these enriched TFs using a 1-tailed Kolmogorov-Smirnov (KS) test of the max ChIP signals (from ENCODE) within genes (as detailed above) of the query gene list compared to the master gene list. A score is calculated using -log of the Benjamini-Hochberg corrected KS p-value x mean of the top 5th percentile query list ChIP values over mean of top (same number as query list) master list ChIP values. The resulting tool performs well against two other TF enrichment tools Enrichr and oPOSSUM-3 based on analysis of three datasets where TFs are knocked out or missing. However, there are a number of recently developed TF enrichment tools including BART, TFEA.ChIP, VIPER, DoRothEA v2 and ChEA3. In fact, in the recent ChEA3 manuscript (<https://academic.oup.com/nar/article/47/W1/W212/5494769>), the authors compare ChEA3 to these TF enrichment tools and MAGICTRICKS (which they call MAGICACT and reference the bioRxiv version of this submitted manuscript). They claim superior performance against all the TF enrichment tools including MAGICTRICKS. Given that the standard in the field is show some kind of advantage against existing top tools and to demonstrate this advantage by not just listing top genes (as is done in this manuscript) but compute performance metrics including precision, recall, AUC, etc (as is done in the ChEA3 manuscript), the following are the major and minor comments/suggested changes.

Major comments:

1. The author(s) should compare MAGICTRICKS to BART, TFEA.ChIP, VIPER, DoRothEA v2 and ChEA3 using performance metrics including precision, recall, AUC, etc and demonstrate some advantage. The perturbed TF datasets used in the ChEA3 manuscript allowed these calculations. These should be presented as main figures in the manuscript.

Firstly, I want to very much thank this reviewer for perhaps the most useful review I have had for any manuscript I have ever submitted. The suggestions and insight offered here have not only massively improved the manuscript but opened my eyes to a whole new world of analysis. I am truly grateful.

The manuscript has been completely re-written to address the points raised above. MAGIC (as it is now called) is compared to CHEA3 using all available libraries, Enrichr and TFEA. Keenan et al already demonstrated superior performance of CHEA to BART, VIPER and DoRothEA. Also, at least in my hands and those of others I asked for assistance, the R scripts for BART, VIPER and DoRothEA were temperamental and ‘buggy’. Thus I chose to focus on the objectively superior performing CHEA3, Enrichr and TFEA.CHIP. I have eliminated any detailed reference to o-Possum. This is because using the ROC and PR metrics, as suggested by the reviewer, for most Factors and datasets, o-Possum is no better than a random classifier. This is discussed in the manuscript.

MAGIC’s output is very dependent on a defensible background list that consists of genes actually expressed in an experiment – as compared to all genes in the genome. Without a background list, MAGIC performs poorly which is the reason that it under-performed in Keenan et al when compared to CHEA3. The dependence on a background list is detailed in Fig 4 of the manuscript. Thus, I have not used the perturbed TF dataset used by Keenan et al because it precludes the use of background lists – none are provided in the dataset. Instead I use 4 datasets and compare the performance of MAGIC to CHEA, Enrich and TFEA. I use a rank analysis to generate D(r)-r curves. I present a Precision Recall and ROC analysis for all algorithms across the four datasets to show that in these real-world tests, MAGIC performs at least as well as the 3 excellent tools.

Again, I thank the reviewer for such constructive comments.

2. ChIP signals for each gene are defined by taking the max within the gene locus. Max is notoriously un-robust. A robust max (95th percentile of the ChIP signal within the gene locus) should also be implemented and available to users.

Based on all the reviewers’ feedback, MAGIC has been completely revamped. The previous version consolidated all CHIPseq tracks for a given factor by taking the max peak seen for that Factor across all tracks in a gene domain. Now, each CHIPseq track is used independently and so no max signal needs to be extracted. The new algorithm has 2312 ChIP-seq tracks covering 684 Factors in 588 cell lines or conditions.

Minor comments:

1. This reviewer would not use a z-score cutoff of 1.65 for tools like oPossum. Something more like a z-score cutoff of 2 or higher should be used when comparing oPossum and MAGICTRICKS.

2. "Kolmogorov" is misspelled on p. 3 of the manuscript.

3. In Fig. 2 legend, "REST" should be replaced by "GATA3, ESR1 and FOXA1".

4. Under Fig. 2, a sentence mistakenly has "...yielded TFs with oPossum at z < 1.65..." which should be "...yielded TFs with oPossum at z > 1.65...".

5. Fig. 3A has "Down in" twice in the text highlighting the CTCF-KO down-regulated gene cluster in the heat map. In Fig. 3 legend, B) should be a "summary output for down-regulated genes" not "up-regulated" as written.

None of the above points carry over to the new manuscript as all the figures are new. All typos are fixed.

Thank you, again, for your comments and time you put into review. The level of your review should be a benchmark against which all reviews should be measured.

Reviewer #3: This manuscript describes MAGICTRICKS, a software tool for predicting factors responsible for the co-regulation of sets of genes. The tool relies on binding profiles from ChIP-seq experiments but, unlike other methods based on experimental data, it does not classify genes as targets and non-targets. Instead, each gene-factor pair is assigned a normalized ChIP-value and the program compares the distribution of values in the list of co-regulated genes and across all genes in the internal database. This is a major innovation that avoids the arbitrary classification of genes into two mutually exclusive categories.

Major comments:
1. As indicated by the author, a major limitation of the tool is that the current input matrix only considers peaks within 5Kb of the gene body. Firstly, aparently this distance was chosen arbitrarily. Second, and more important, many transcriptional regulators preferentially bind distant enhancers and thus most of theirs targets won’t be represented in the matrix. A potential solution to assign distant peaks to specific genes, is using the experimental evidences recorded in databases such as GeneHancer.

Based on this reviewer’s excellent comments, MAGIC has been extensively modified and I thank the reviewer for providing the impetus to greatly improve the algorithm. The previous version had an arbitrary gene window of 5Kb either side of the gene body. The current version offers the user a choice of 6 different matrices for analysis:

1Kb_gene.mtx

1Kb_gene_with_engineered_factors.mtx

1Kb_promoter.mtx

1Kb_promoter_with_engineered_factors.mtx

5Kb_gene.mtx

5Kb_gene_with_engineered_factors.mtx

Thus, the user can now use matrices containing binding data of Factors within a 5Kb window of the gene body, 1Kb window of the gene body, 1Kb of the promoter and with or without ChipSeq tracks from engineered (eg CRISPRed) Factors.

The current version of the MAGIC does not utilize distal enhancer information but work is in progress to incorporate data from GeneHancer as the reviewer suggests. Incorporation of such data turned out not be trivial, but I anticipate have new matrices to add to the above by end of this summer.

2. A second limitation is the relatively small set of Factors and Regulators included in MAGICTRICKS. The author could consider using ReMap instead of just data from the ENCODE project.

The new algorithm uses the latest ENCODE dump that has 2312 ChIP-seq tracks covering 684 Factors in 588 cell lines or conditions. Unfortunately the ReMAP CHIP seq data is binary in nature so cannot be incorporated into MAGIC.

3. The selection of the highest ChIPseq value from a given factor across cell lines supposedly maximizes the likelihood that the site is universal, i.e. present in other cell types not represented in the original datasets. This hypothesis should be supported by references or data. In addition, this approach will blur tissue-specific or treatment-specific responses. Would not it be better to keep each experiment as an independent column in the internal matrix?

This is exactly what is now been incorporated and I thank the reviewer for this excellent suggestion.

4. The examples used to illustrate the performance of MAGICTRICKS, although convincing, are just individual cases and thus do not provide compelling evidence for the general performance of the method. The use of complete collections of well-defined transcriptional signatures or gene sets, such as those in the GSEA MSigDB, will probably be more appropriate.

MAGIC’s output is very dependent on a defensible background list that consists of genes actually expressed in an experiment – as compared to all genes in the genome. Without a background list, MAGIC performs poorly which is the reason that it under-performed in Keenan et al when compared to CHEA3. The dependence on a background list is detailed in Fig 4 of the manuscript. Thus I have not used the perturbed TF dataset used by Keenan et al or MSIGDB gene sets because it precludes the use of background lists – none are provided in the dataset. Instead I use 4 datasets and compare the performance of MAGIC to CHEA, Enrich and TFEA. I use a rank analysis to generate D(r)-r curves. I present a Precision Recall and ROC analysis for all algorithms across the four datasets to show that in these real-world tests, MAGIC performs at least as well as the 3 excellent tools.

5. The Score for each transcription factor integrates the statistical significance of the KS test and a parameter (r) that tries to represents the size effect. Although we agree it is important to include both factors, the value of r is based on few extreme data points and, as such, it is not a indicators of the overall difference between master and query lists CDFs. We suggest the use of another parameters to represent the size effect (difference between both distributions) such as Ds-Di or the difference between the c value required to reach 0.5 of the CDF.

I thank the reviewer for the suggestions. I tried using Ds-Di, D, differences in chip between the background and query to get 50% of CDF and none were as discriminating as using the mean of the extreme datapoints. In my experience, a typical gene list entered into MAGIC will be on the order of hundred to many hundred genes. Thus r will be based on at least 5 genes and often many 10s of genes. In the next version of MAGIC, I will allow the user to play with this value and increase the fraction of extreme genes to use as a measure of effect size.

6. The tool is implemented for two specific operating systems. A cross-platform tool would be more widely accepted.

Currently, MAGIC will work on Windows machine or any machine with a Darwin based OS. I am in the process of moving to a web-based tool which will be cross platform. Also, once the code is ‘cleaned and beautified’, I will deposit on Github.

Minor comments:
1. The programming language used for the implementation is not stated.

This is now fixed in the Methods section. I state “*MAGIC was written in Python 3.7 and packaged into a stand-alone executable using Pyinstaller 3.6.*“

2. Supplementary file 9 is not referenced in the manuscript.
3. There is a typo in the paragraph before the last in the Results section. It should say “supplemental file 12-Enrich tab” instead of “supplemental file 1-Enrich tab”
4. In the analysis of the CTCF knock-out experiment the conversion of mouse to human IDs is not explained.
5. The captions for figures 3B and 3C contradict the main text.

These issues no longer apply to the current version of the manuscript.

*In summary I want to thank all three reviewers for the most useful, insightful and enjoyable review process I have ever been part off. It was a real pleasure working through your ideas, comments and suggestions. I wish you the very best in your future research*.
