# Supplementary material for: MAGIC: A tool for predicting transcription factors and cofactors driving gene sets using ENCODE data
Source: PLoS Comput Biol. 2020 Apr 6;16(4):e1007800. doi: 10.1371/journal.pcbi.1007800 (PMC7162552; doi:10.1371/journal.pcbi.1007800)
Supplement: Attachment — Submitted filename: Response_to_Reviewers_II.docx [file pcbi.1007800.s015.docx]

Reviewer #1: The revised manuscript has been greatly improved, it is now clearer and addressed all my concerns. The new corrected algorithm accepts more parametrisations and I expect the authors will continue its development. I hope the authors will release updates that will allow to switch the TFs&factors analysis from genes to transcripts and splicing pattern identification using an adequate background file.

Thank you! I expect a new matrix option that uses transcripts (often multiple per gene) rather than genes will be ready by summer this year.

------
Reviewer #2: Dr. Roopra has addressed all my comments/suggestions in this revised manuscript.

There are just a few remaining minor issues that need to be addressed:

1. In addition to being made available from a University of Wisconsin website, MAGIC should be made available on GitHub, which serves as a more permanent site.

The Python script will be deposited at GitHub once I have ‘cleaned it up’ a little. The packaged app itself cannot be hosted at GitHub due to size limitations. I expect the script to be uploaded within the next 30 days.

2. In the Methods section under the Precision Recall... subsection, "FET" should be defined as "Fisher's Exact Test" and there should be a period after "...each test (Calls Vectors)".

I have edited accordingly – thanks for catching these errors!

3. In the Methods section under the Rank Analysis subsection, D(r) is more clearly defined in the Results section as cumulative of fractional ranks for the manipulated Factor and PIs. I would use this definition in the methods section as well.

I agree that the wording in the results section is clearer. I have replaced the wording in the methods section to now resemble the text in the results section.

4. In the Methods section under the Rank Analysis subsection, the sentence "Blocks of positive calls in the Calls Vector were all assigned the rank of the first element of the block." is unclear. How are "blocks" defined? I was under the impression that every positive call was ranked.

Every positive call is indeed ranked. I have modified the text to more clearly explain how the D(r)-r plots are generated. Specifically, and only for, the D(r)-r figures, “*contiguous runs of positive calls (a block of positive calls) are assigned the rank of the first element in the block*.” This is done because a Factor is deemed positive if it appears in the list of PIs (Potential Interactors) which itself is unranked. Thus if an algorithm produces a contiguous list of 10 positive Factors, followed by a run of 10 negatives (missed calls) and then a run of 10 positives, all 10 Factors in the 1^st^ block are given rank = 1, all 10 factors in the second positive block are given rank = 21. This is my attempt allow each algorithm to highlight a block of meaningful calls.

5. In the Methods section under MCF7 RNA..., the last sentence is missing a period after "...(S1 Table)".

Corrected.

6. In the Methods section under Implementation of MAGIC in the second paragraph, "...each query list and generate a series..." should be "...each query list and generates a series...".

Corrected.

7. In the Results section in the Identification of Cofactors...subsection, "...either high or low ranks would produce a uniform distribution (which equates to r)" should be "...either high or low ranks would produce a uniform p-value distribution (which equates to r)" for clarity (unless something else was meant). it's known that a null model or random data with no enrichment will produce a uniform p-value distribution.

Corrected.

8. In the Discussion section, "...depending on the set of genes in it and so the maximal..." should be "...depending on the set of genes in it, and so the maximal...".

Corrected

Best,
Stefan Bekiranov

-----

Reviewer #3: The author have addressed most of our concerns and the current version of the method is clearly improved as compared to the original one. However, some concerns regarding the manuscript still remain, as detailed below.

Major comments:
1. The number of cases used to illustrate the performance of MAGIC have been slightly increased but, given the limited number of datasets and the arbitrary criteria for their selection, they still do not provide compelling evidence for the general performance of the method. At least the author should clearly acknowledge this important limitation when drawing conclusions. Particularly, when comparing MAGICTRICKS with the rest of tools included in the manuscript as reference.

I have added the following statement to the discussion to address this very important caveat to the analysis:

“MAGIC was tested against CHEA3, TFEA and Enrichr on 4 datasets where it performed well. However, the general performance level of MAGIC against the leading algorithms will only be truly revealed with significantly increased numbers of tests.”

2. The criteria to select genes of interest should be the same across the experiments used for validation. Even if the experiments weren't homogeneously process from reads to diferential expression. The author should provide a robust reason to not follow the same criteria when selecting genes for testing.

The reason for using different criterion for selecting DEGs across datasets was to try and emulate ‘real world’ scenarios. Thus using the same ‘fold cutoff’ or other such parameter results in vastly different numbers of DEGs and I aimed to keep the numbers of genes in the hundreds of genes range rather than a few tens or thousands.

Additionally, the author stated "Genes up-regulated more than 3 Standard Deviations from the mean fold change....". Since Fold Change doesn't follow a simmetric distribution, this apporach only serves to select up-regulated genes. Did the author meant the log-fold change?

Thanks for pointing that out! I did indeed mean log-fold and have corrected the manuscript accordingly.

3. The manuscript indicates that the tool is implemented for two specific operating systems, yet the address <https://go.wisc.edu/MAGIC>. only contains stand-alone for MacOS. More importantly, since the tool is written in python, the author could provide the source code so it can be used in any platform.

Thanks for pointing this out. I pulled the Windows version soon after resubmitting the manuscript as there was a file handling bug in it. I have now corrected that and will be packaging and hosting the Windows version over the next week.

4. page 19 states that "plasticity[18]. 432 genes were down-regulated upon CTCF loss out of 17,015 expressed genes (S3 Table).". However, according to the results reported on GEO, there are 321 genes with log2(FC)<0 and adjusted p-value <0.05. If the author has analized the RNA-seq following a different pipeline that the one reported on GEO, it should be stated.

I used a different pipeline from GEO and that is now clarified in the text. Thus “A gene with an RPKM ≥1 in all controls or all CTCF KO cells was considered for further analysis and fold changes, statistics and gene lists were then generated as above for REST knock-down cells. 17,015 genes were expressed by this criterion with 432 genes down-regulated at least 1.5 fold with a corrected p<0.25”

5. On page 23 the author states "Nevertheless, it provided an extremely powerful prediction of EZH2 induction which was missed by CHEA3 and TFEA but was confirmed
by Enrichr.". However, we analyzed the dataset provided as supplementary information in reference 43 (<https://journals.plos.org/plosone/article?id=10.1371/journal.pone.0226733#sec033>) and found that TFEA identifies EZH2 (along with SUZ12 and JARID2) as the top candidate for this dataset. Moreover, five of the top ten datasets represent EZH2 ChIPseqs. In the case of ChEA3 (literature library), the top candidate is MTF2 (also known as Polycomb-Like 2) that mediates binding of PRC2 to DNA. The author should correct their conclusion regarding the performance of TFEA and ChEA3 or provide a compelling argument to explain the indicated discrepancies.

I have removed the offending statement. It was a remnant of a much older version of the manuscript which was using an older pipeline to generate DEG lists for the PLoS One paper. Thanks for bringing that to my attention!

Minor comments:
1. It's possible to ask ReMap's authors for the MACS2 outputs of their ChIP collection. The current ENCODE dump has a wide range of TF covered, but you might want to consider ReMap for a future update.

Thanks for the advice – I will indeed contact the MACS2 authors and provide updated matrices.

2. Methods. Page 15. "An emperical distribution function...", should say "empirical".

Corrected

3. Figure legends page 24, spelling of "reciprical"

Corrected

3. Last line pag 16 says "ChIP value at dsup", for consistency "dsup" should by capitalized as "Dsup"

Corrected

4. figure 1, please indicate the meaning of "ND".

“ND = Not Determined; Factor not present in library.” This is now added to the legend.

5. Quality of figures 2 and 3 is poor.

The highest resolution TIFF files will be used for subsequent upload.
----------------

Finally, I would like to re-iterate what I said in my previous response to reviewers: This process has been one of the most enlightening and enjoyable manuscript submissions I have ever been part of. It has been a real joy and an honour to interact and learn from the reviewers’ expertise.

Most grateful thanks to all three reviewers for a most scholarly interaction.

Avtar
